# Quantum Coherent Feedback Control for Generation System of Optical Entangled State

**DOI:** 10.1038/srep11132

**Published:** 2015-06-05

**Authors:** Yaoyao Zhou, Xiaojun Jia, Fang Li, Juan Yu, Changde Xie, Kunchi Peng

**Affiliations:** 1 State Key Laboratory of Quantum Optics and Quantum Optics Devices, Institute of Opto-Electronics, Shanxi University, Taiyuan 030006, P. R. China

## Abstract

The non-measurement based coherent feedback control (CFC) is a control method without introducing any backaction noise into the controlled system, thus is specially suitable to manipulate various quantum optical systems for preparing nonclassical states of light. By simply tuning the transmissivity of an optical controller in a CFC loop attached to a non-degenerate optical parametric amplifier (NOPA), the quantum entanglement degree of the output optical entangled state of the system is improved. At the same time, the threshold pump power of the NOPA is reduced also. The experimental results are in reasonable agreement with the theoretical expectation.

In recent years, quantum control theory has been widely used in various protocols of quantum optics research^1^,^2^,^3^,^4^,^5^. The control methods are usually divided into two kinds: measurement-based feedback control^1^,^2^,^3^ and non-measurement based coherent feedback control (CFC)^4^,^5^. CFC is a control approach which never introduces any backaction noise into the controlled system^6^,^7^,^8^,^9^,^10^, and thus it extensively attracts interest since the initial concept was proposed by H. M. Wiseman *et al.*^4^. R. Hamerly *et al.* pointed out that CFC could outperform the best possible linear quadratic Gaussian measurement-based schemes in the quantum regime of low steady-state excitation number^6^. A CFC system for testing basic principles of linear quantum stochastic control theory was experimentally realized by H. Mabuchi^7^. The simple and general algebraic methods for describing a series of connections in quantum networks were presented^11^. J. Kerckhoffhave *et al.* designed a novel approach for the quantum error correction based on CFC^12^.

Nonclassical states of light, such as squeezed and entangled states are important resources for the fundamental study of quantum optics and continuous variable (CV) quantum information processing^13^,^14^,^15^,^16^. It is necessary to find effective schemes of manipulating quantum states of light. Firstly, the manipulation of optical nonclassical states was realized by the phase-sensitive operations. G. S. Agarwal theoretically studied the interferences of the quantum fluctuations from a cavity driven by a quantized squeezed state of light^17^ and then the quantum manipulation phenomenon was experimentally demonstrated by injecting a squeezed state of light into an optical cavity^18^. Successively, the phase-sensitive manipulation of entangled state of light was theoretically proposed and experimentally realized^19^,^20^,^21^. Because there is no measurement element in CFC, i.e. no the access noise, the CFC is a excellent approach to implement the manipulation of the noise-sensitive optical squeezed or entangled states. The enhancement of squeezing of optical field based on CFC was proposed and experimentally realized, which proved the applicability of the CFC in nonclassical regime^11^,^22^,^23^,^24^,^25^. Then a scheme of manipulating the multipartite entangled states of light through CFC was theoretically proposed and detailedly analyzed by our group^26^. Very recently, the CFC feedback-optimized extraordinary optical transmission of CV entangled states through a hexagonal metal-hole array was experimentally realized^27^. In this paper, we present the experimental demonstration of the theoretical proposal^26^. The experiment shows that the entanglement degree of the entangled state produced by a non-degenerate optical parametric amplifier (NOPA) can be manipulated by a tunable beam splitter in CFC loop and the entanglement up to −6.0 *dB* is obtained. Especially, the threshold pump power of the NOPA is decreased and thus the same entanglement can be achieved under a relative lower pump level for a given NOPA. The experimental measurements reasonably agree with the theoretical calculations. The advantages and the restrictions of the CFC scheme are discussed.

## Results

### Schematic of CFC-NOPA system

**Figure 1:**
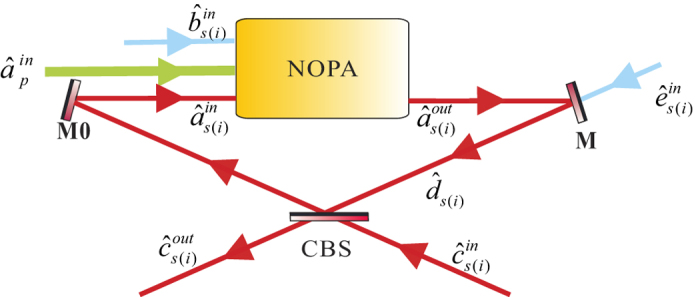
The principle schematic of CFC-NOPA system. The pump field 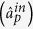 and a weak signal (idler) beam 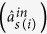 are coupled into the NOPA. The output entangled state 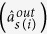 from the NOPA is injected in a CFC control loop. The output optical field 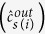 of the system is made up of two parts, one part is the reflected input field of the CBS and the other part is the transmitted field of the optical mode 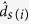 in the CFC loop.

The schematic of the system is shown in Fig. 1. The NOPA is an optical cavity involving a type-II phase matching nonlinear crystal. The pump field 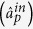 at the harmonic-wave frequency (2*ω*) and a weak signal (idler) beam 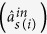 at the subharmonic-wave frequency (*ω*) are coupled into the NOPA. Via an intracavity frequency-down conversion of the pump field inside the NOPA, the produced two nondegenerate subharmonic-wave modes form an Einstein-Podolsky-Rosen (EPR) optical entangled state with quantum correlations of both amplitude and phase quadratures^28^,^29^,^30^. The output entangled state 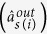 from the NOPA is injected in a CFC control loop composed of two mirrors (M and M_0_) and a control beam splitter (CBS) with the transmissivity T at *ω*. The total extra loss in the CFC loop is regarded as a vacuum noise 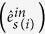 coupled from the mirror M with a transmissivity L at *ω*. The input signal (idler) mode of the entire CFC-NOPA system is a weak optical field of coherent state at *ω* 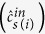 which is coupled into the system from CBS. The output optical field 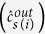 of the system is made up of two parts: one part is the reflected input field of the CBS 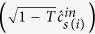 and the other part is the transmitted field of the optical mode 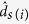 in the CFC loop 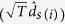
26. The transmissivity of the input coupler of the NOPA for the pump field and the subharmonic optical field are expressed by γ_10_ and γ_1_, respectively. All the unwanted other losses in NOPA 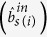 can be thought as the intracavity losses of the signal (idler) mode (γ_2_) and the pump field (γ_20_), respectively.

### Theoretical analysis of the pump threshold and correlation variances of CFC-NOPA system

The quantum Langevin motion equations of the pump optical field 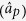 and the parametric down-conversion optical field 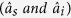 when the NOPA and the CFC loop resonate are^31^,^32^,^33^:




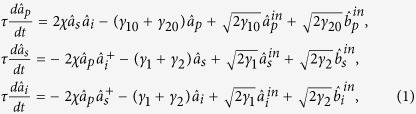




where 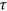 is the round trip time of light in the NOPA, χ is the parametric coupling constant of the crystal. In the linearized description of fields, the operators 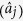 can be expressed by the sum of an average steady state value (*a*_*j*_) and a fluctuating component 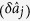, that is, 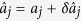, (*j* = *p*, *s* and *i*). From the stationary equation and the relationship between the input and output field of the NOPA system^31^, the oscillation threshold (*P*_*th*_) of the CFC-NOPA can be easily calculated:




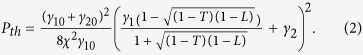




**Figure 2:**
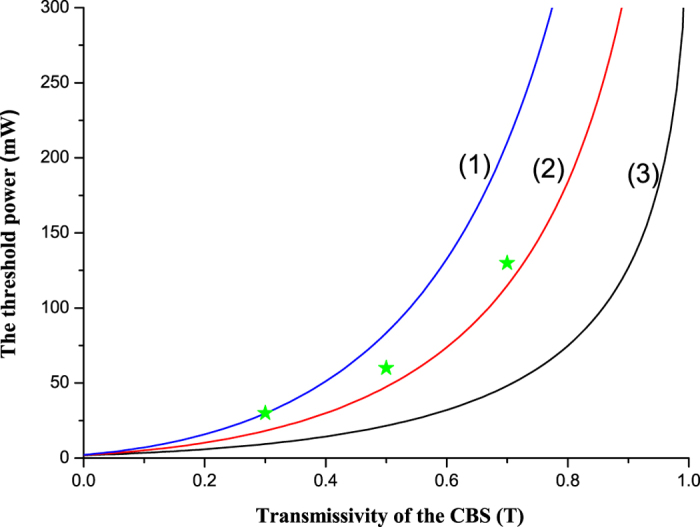
The dependence of the threshold of the CFC-NOPA cavity on the transmissivity of CBS for subharmonic optical field. The traces (1) (2) and (3) are the calculated threshold pump power when γ_1_ is selected as 0.07, 0.05 and 0.03, respectively. The star is the experimentally measured result when γ_1_ = 0.05.

The dependence of the threshold of the CFC-NOPA system on the transmissivity of CBS (*T*) is shown in Fig. 2. The trace (1) (2) and (3) are the calculated threshold pump power when γ_1_ is selected as 0.07, 0.05 and 0.03, respectively. It can be seen that the threshold of the CFC-NOPA system reduces a lot comparing with the NOPA without the CFC control (*T* = 1). When *T* is 1, the CFC-NOPA system works as an usual NOPA. If *T* < 1, the CFC loop feeds back a part of its output optical field into the NOPA. The lower the *T* is, the stronger the intensity of the feedback light is. Since the feedback subharmonic-wave field increases the intracavity intensity of the signal and idler modes in the NOPA, its threshold pump power for the frequency-down conversion is reduced naturally.

The amplitude quadrature 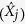 and the phase quadrature 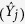 of the optical mode 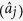 equal to: 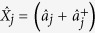 and 
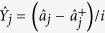
, respectively. According to the relationship between the input and output field of NOPA^31^ and Eq. (1), the correlation variances of the quadrature amplitude and quadrature phase of the output field for the CFC-NOPA system are calculated:




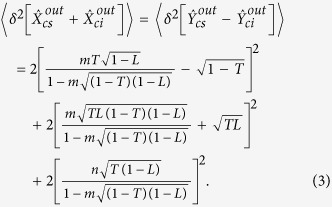




where, *m*=(−*κ* + γ_1_ − γ_2_ − *i*Ω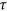)/(*κ* + γ_1_ + γ_2_ + *i*Ω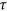); 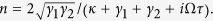 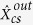 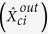 and 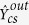 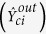 are the quadrature amplitude and quadrature phase of the output signal (idler) mode 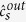 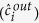, Ω is the analysis frequency and *κ* stands for the non-linear conversion coefficient which is related to the parametric coupling constant χ^19^. In the calculation, we have assumed that the signal and the idler modes are totally balanced, which is easily realized in usual experiments.

### Experimental setup and results

**Figure 3:**
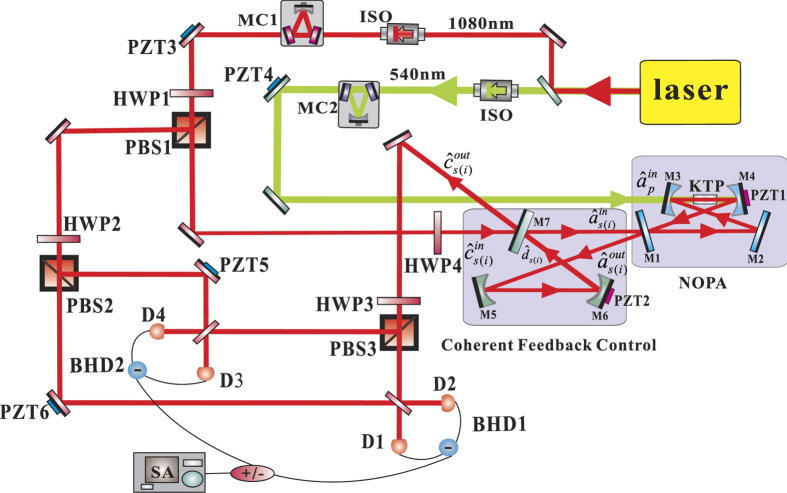
Experimental setup of manipulating and enhancing the EPR entangled states with the CFC loop. Laser: Nd: YAP/LBO laser source; MC1: Mode cleaner of 1080 *nm*; MC2: Mode cleaner of 540 *nm*; HWP1-4: λ/2 waveplate; PBS1-3: Polarizing beam splitter; ISO: Isolator; PZT1-6: Piezoelectric transducer; BHD1-2: Balanced homodyne detector ; SA: Spectrum analyzer.

Figure 3 shows the experimental setup. The laser source is a solid-state single-frequency and stable-frequency continuous wave Nd: YAP/LBO laser with two output wavelengths at 540 *nm* and 1080 *nm* (CDPSSFG-VIB made by YuGuang company). The maximal output power are 4 watt for 540 *nm* and 1 watt for 1080 *nm*, respectively. The two output lasers are separated into two parts by a dual-color mirror with a high-reflection coated for 1080 *nm* and an anti-reflection coated for 540 *nm*. The mode cleaner (MC1) is a three-mirror ring cavity that provides the spatio-temporal filtering of the 1080 *nm* laser for the downstream experiment and is used as the reference cavity for stabilizing the frequency of the laser source also. The finesse of the MC1 is 500 for 1080 *nm*. The mode cleaner (MC2) which has the identical structure with MC1 is used for reducing amplitude and phase fluctuations of the pump field and its finesse is 650 for 540 *nm*. The lengths of MC1 and MC2 are servo-controlled to resonate with their carrier field by lock-in amplifiers. The cleaned pump laser (540 *nm*) is injected into the NOPA cavity from M3 as the pump field. One part of the cleaned infrared laser (1080 *nm*) serves as the local oscillators of the balanced homodyne detectors (BHD1 and BHD2) and the other part is used as the seed beam of the CFC-NOPA system. The NOPA cavity is a four-mirror ring cavity consisting of two plat mirrors (M1, M2) and two spherical mirrors (M3, M4). A *α*-cut type-II phase matching KTP (KTiOPO_4_, potassium titany1 phosphate) crystal is placed between M3 and M4. The plat mirror M1 is used as the input and output coupler of the NOPA, which is coated with anti-reflection of the pump field 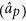 and partial transmission of the subharmonic optical field 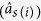 with a designated transmissivity of γ_1_ = 0.05. The mirrors (M2, M3, M4) are all coated with highly reflecting of the subharmonic modes and anti-reflecting of the pump field. The CFC loop is also a four-mirror ring cavity which includes two plat mirrors (M1, M7) and two spherical mirrors (M5, M6). The transmissivity T of the plat mirror M7 for the subharmonic optical field is changeable and M7 is used as the controller of the CFC-NOPA system. M5 and M6 are highly reflecting for the subharmonic optical field. The radius of curvature of all four spherical mirrors (M3, M4, M5, M6) equals to 100 *mm*. The type-II non-critical phase matching KTP crystal of dimensions 3*3*10 *mm*^3^ is placed in a copper-made oven and is temperature-controlled around 63 °*C* with a peltier element for achieving the optimal phase matching. M4 is mounted on a piezoelectric transducer (PZT1) to scan actively the cavity length of the NOPA or lock it on the resonance with the injected signal as needed by the Pound-Drever-Hall technique. The geometric length and the waist size of the NOPA cavity are 540 *mm* and 62.8 *μm*, respectively. In order to effectively manipulate the EPR entangled states generated by the NOPA, PZT2 is mounted on M6 to actively scan the cavity length of CFC loop.

**Figure 4:**
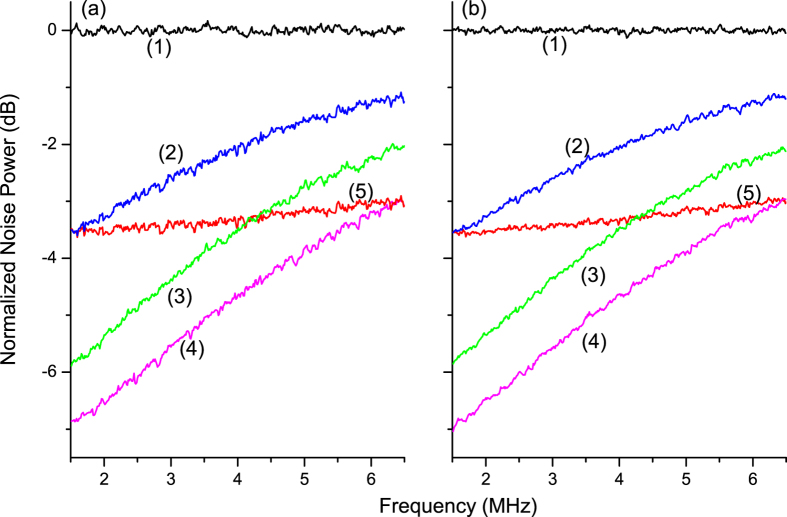
The measured amplitude-sum (**a**) and phase-difference (**b**) correlation variances noise powers of the output beams, where (1) is the normalized QNL; (2–5) is the correlation noise powers with T = 0.31, 0.50, 0.72 and 1.00, respectively. The measurement parameters of SA: RBW 10 kHz; VBW 100 Hz.

The input coherent optical beam at *ω* 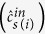 is equally distributed into two orthogonal intrinsic polarizations of the type-II phase matching KTP by a λ/2 wave plate (HWP4) placed ahead of CBS (M7 in Fig. 3). The two orthogonally polarized optical beams serve as the seed beams of the subharmonic-wave signal and idler modes in the NOPA, respectively. The measured threshold power of the CFC-NOPA is 30 *mW*, 60 *mW*, 130 *mW* and 1150 *mW* for *T* = 0.3, 0.5, 0.7, 1, respectively, which is marked by a star in Fig. 2. It can be seen that the measured threshold power of the CFC-NOPA cavity is a little higher than the theoretically calculated value, that is because some extra loss in the experimental system is not accounted in Eq.(2). In the experiment, the power of the injected subharmonic-wave is kept at 200 *μW*. When the transmissivity of CBS is chosen as *T* = 0.7 and the pump power is 60 *mW*, the classical gain of 4 times is observed if only scanning the length of NOPA. However, if the lengths of both NOPA and CFC loop are scanned simultaneosly, the maximal classical gain of the NOPA can reach to 35 times. Locking the relative phase between the pump field and the injected seed optical beam to (2*n* + 1)*π* (*n* is an integer), the NOPA operates at the state of deamplification. In this case, the EPR entangled states of light with the anti-correlation of the quadrature amplitudes and the correlation of the quadrature phases are generated^20^. The output entangled beams with orthogonal polarizations are separated by a polarizing beam splitter (PBS3) and then detected by two sets of BHDs (BHD1 and BHD2), each of which consists of a 50/50 beam splitter and two high quantum efficiency InGaAs photo diodes. Meanwhile, we lock the relative phase between the local beam and the injected signal (idler) to 0 or *π*/2 to measure its amplitude or phase quadrature by BHD1 (2), respectively. The noise powers of the amplitude or phase quadratures measured by BHD1 and BHD2 are combined by the positive (negative) power combiner (+/−) and then the correlation variances of the amplitude-sum (phase-difference) are measured by a spectrum analyzer. The measured noise power spectra of the amplitude-sum (a) and the phase-difference (b) between the output signal and idler optical fields from 1.5 *MHz* to 6.5 *MHz* are shown in Fig. 4. Trace (1) is the QNL, which is obtained by blocking the output beam of the CFC-NOPA. Firstly, we measure the entangled degree of the EPR entangled beams directly generated by the NOPA through changing the transmissivity of CFC controller (M7) to *T* = 1.00 i.e, the CFC loop does not work. The measured correlation variances of the amplitude-sum and the phase-difference are about −3.5 *dB* below the corresponding QNL as shown in trace (5) in Fig. 4. By means of replacing the mirror M7 with mirrors having different transmissivities, the controller CBS (M7) can be tuned to *T* = 0.72, 0.50 and 0.31, respectively. When the cavity length of the CFC loop is swept by PZT2, the correlation variance of the output beams from the CFC-NOPA fluctuates rapidly, and the entanglement even disappears sometimes. To realize the stable entanglement enhancement and manipulation, the cavity length of CFC loop is locked to resonate with the injected seed beams during the experiment. Traces (2–4) in Fig. 4 are the measured correlation variances of the amplitude-sum and the phase-difference corresponding to the *T* = 0.31, 0.50 and 0.72 respectively. We can see that the optimal entanglement enhancement appears at lower analysis frequency as theoretically expected^26^. Because the cavity bandwidth of CFC-NOPA is smaller than that of NOPA. The delay of the light in the feedback loop will affect the control performance at some extent, the influence becomes stronger at the region of higher frequencies, so the performance of entanglement enhancement of the CFC is limited by the bandwidth of the CFC-NOPA system, which results in that the correlation variances of output beams from the CFC-NOPA are higher than that of the beams coming directly from the NOPA at higher frequencies. For the optimal transmissivity (0.72) of CBS (trace (4) in Fig. 4), the entanglement is no longer increased by the CFC and some extra noises deriving from the CFC will raise the correlation variances of the output field from the NOPA when the frequency is higher than 7 MHz. The entanglement of the output optical field can be manipulated by tuning the transmissivity of CBS. From Fig. 1, we can see that both the output optical field of the CFC-NOPA system and the field fed back into the NOPA include two parts. One part is the EPR entangled state of light, which plays the positive role for the entanglement enhancement. The other part is input coherent optical field and the extra vacuum noise resulting from the CFC loop, which reduces the entanglement and thus plays the negative role for the entanglement enhancement. When T is lower, the coherent light directly reflected by CBS increases and the positive role of the entanglement enhancement possibly becomes smaller than the negative influence of the input coherent light, which results in the quantum correlation variances of the output field of the CFC-NOPA system being higher than that without using the CFC loop. For the given system, the optimal entanglement enhancement is obtained at *T* = 0.72, which agrees with the theoretical expectation from Eq.3.

## Discussion

For the conclusion, we design and experimentally demonstrate a CFC-NOPA linearly optical system for the generation and manipulation of optical entangled state. Not only the entanglement between the output optical modes can be enhanced but also the threshold pump power of the NOPA can be reduced under appropriate conditions. By simply adjusting the transmissivity T of the CBS in the CFC loop, the quantum feature of the output entangled state can be manipulated. The presented CFC scheme based on linear optics is able to be applied in other systems for the generation and control of the entangled states, such as nonlinear optical fiber or nanophotonic devices^34^,^35^,^36^,^37^.

## Additional Information

**How to cite this article**: Zhou, Y. *et al.* Quantum Coherent Feedback Control for Generation System of Optical Entangled State. *Sci. Rep.* **5**, 11132; doi: 10.1038/srep11132 (2015).
